# Short communication: A practical farm-based trial to compare ewe nematode control strategies in peri-parturient ewes

**DOI:** 10.1371/journal.pone.0236143

**Published:** 2020-08-13

**Authors:** Charlotte L. Kerr, David R. Armstrong, Alison J. Anderson

**Affiliations:** 1 Department of Infectious Disease Epidemiology, Faculty of Epidemiology and Population Health, London School of Hygiene and Tropical Medicine (LSHTM), London, United Kingdom; 2 Zoetis UK, Springfield Drive, Leatherhead, United Kingdom; Tokat Gaziosmanpasa University, TURKEY

## Abstract

The focus of gastro-intestinal parasite control in the sheep industry is increasingly on finding a balance between maintaining productivity of the flock whilst minimising selection for anthelmintic resistance to preserve anthelmintic efficacy for the future. Periparturient ewes represent the major source of gastro-intestinal parasites for growing lambs and are therefore a priority for parasite control. This study examines the impact on ewe faecal egg counts (FECs), lamb FECs, lamb daily live weight gains (DLWGs) and pasture larval counts of treating groups of ewes two weeks prior to lambing with either, a long-acting moxidectin treatment, short-acting doramectin or control. Six groups of twenty ewes were allocated to individual paddocks, two groups allocated to each treatment, and weekly faecal sampling was performed throughout from the ewes and from six weeks after the start of lambing in the lambs. Treatment group was found to have a significant effect on both ewe FEC (p<0.001) and lamb FEC (p = 0.001) with the group receiving the long-acting anthelmintic having the lowest ewe and lamb FECs. There was no significant effect on the DLWGs of the lambs. Pasture larval counts at the end of the study period were lowest in the long-acting wormer treatment group. The use of long-acting moxidectin may be helpful as part of a parasite control programme by reducing the worm burdens of ewes and their lambs, decreasing the number of anthelmintic treatments required in that year and by reducing pasture contamination for those sheep which will graze the pasture in the next year. However, like all anthelmintics, its use should be judicious to avoid selection for resistance.

## 1 Introduction

It is well recognised that ewes represent the major source of infection by gastro-intestinal parasites for growing lambs due to the periparturient relaxation in immunity (PPRI) they experience in late gestation and early lactation [1]. This results in increased worm burdens and a consequent contamination of pasture which the lambs graze. Historically, deworming of all ewes prior to lambing was considered a good measure of controlling this. However, resistance to benzimidazoles is now ubiquitous in the UK and the presence of strains resistant to imidathiazoles and macrocyclic lactones is also increasingly seen, as well as multi-drug resistant parasites [2–7]. Blanket worming of all sheep is deemed to be a risk factor for the selection of resistance against anthelmintics and more judicious/considered strategies are required [8].

Studies assessing productivity impacts of parasite burdens in sheep found that gastrointestinal nematodes may be responsible for a 15% reduction in weight gain, as well as costing the UK sheep industry over £80 million every year, more than any other endemic disease [9, 10]. With low margins in the UK sheep industry, uncontrolled parasite burdens can easily mean the difference between profit and loss [11]. Not only does it impact on farm economics it also impacts on the health and welfare of the UK sheep flock causing both morbidity and mortality.

Short-acting anthelmintic treatments, including benzimidazoles, imidathiazoles and avermectins, given prior to lambing are unlikely to provide protection over the whole period of PPRI, resulting in a rise in worm burden during mid-lactation and subsequent pasture contamination. The use of long-acting anthelmintics as a means of controlling egg output by ewes throughout the entirety of the PPRI period has thus been proposed as an alternative to short acting treatment [16]. Currently around half of all pre-lambing treatments given are anthelmintics without persistent action (personal comms.).

This study compared the effect of three different ewe treatments, a long-acting moxidectin dewormer, a short-acting doramectin dewormer and no deworming treatment, received two weeks prior to the start of lambing on ewe and lamb worm burdens, pasture larval counts and lamb growth rates. The findings of this study will inform effective strategies to combat PPRI, which not only minimise sheep parasite burdens but also promote responsible anthelmintic use.

## 2 Methods

### 2.1 Study population

The study was conducted at FAI farm in Wytham, Oxford. The farm is a mixed enterprise operating under organic farming standards based on the Oxford University farming estate, and has a breeding flock of 1300 homebred Coopworth and Easycare woolshedder ewes. Lambing commences on 6th April 2017, with the majority of the flock lambing outdoors. One hundred and twenty outdoor-lambing twin bearing ewes were recruited into the trial. The trial began 2 weeks prior to lambing, and concluded at weaning of the lambs (1^st^ August 2017).

Ewes were recruited into the study on the basis of fecundity (twin-bearing), lambing cycle (served in the first cycle therefore due to lamb within the first 3 weeks of lambing) and health status, with forty ewes randomly assigned to one of three treatments: treatment 1 (SA: short acting wormer, doramectin 10mg/ml, dose rate of 1ml/50kg), treatment 2 (LA: long acting wormer, moxidectin 20mg/ml LA, dose rate of 0.5ml/10kg) and a control (C: untreated) group. Each treatment group was divided in two to provide a duplicate. All ewes were health checked and body condition scored at the start of the study. All ewes recruited into the study were body condition score 3 or greater and Coopworth type ewes. One field of permanent pasture was divided into six equal paddocks using electric fencing and each paddock was provided with a water source. Each paddock was randomly allocated to a treatment group and was populated with 20 ewes from that treatment group. The number of ewes in each paddock was aligned with the standard on-farm stocking density of three ewes per acre. Two weeks prior to lambing the ewes were faecal sampled, given their assigned treatment and populated into their paddocks. The short-acting wormer, doramectin 10mg/ml (Dectomax 10mg/ml solution for Injection for Cattle and Sheep, Elanco, UK) was given at a dose of 1ml per 50kg by intramuscular injection. The long-acting wormer, moxidectin 20mg/ml LA (Cydectin 20mg/ml LA Solution for Injection for Sheep, Zoetis, UK), was given at a dose of 0.5ml per 10kg by subcutaneous injection at the base of the ear. All ewes were weighed prior to treatment to ensure adequate dosing. Ewes were removed from their assigned paddock if they lost a lamb or were unable to mother two lambs. This was done to make sure all ewes were under the same level of lactational pressure. There was a particular problem with predation by foxes resulting in the loss of a number of lambs. Fox lights were instituted once predation was noted. The number of ewes and lambs present on the last day of the trial in each paddock is shown in Table 1.

**Table 1:** Number of ewes and lambs remaining in each paddock when ewes were shorn.

Ewe Group	Treatment	Number of ewes	Number of lambs
A	Control	17	34
B	Control	16	32
C	LA	18	33
D	LA	17	32
E	SA	12	24
F	SA	19	38

### 2.2 Data collection and analysis

From two weeks prior to lambing to shearing of the ewes, the ewe groups were faecal sampled weekly, taking a proportional number of samples per field for the number of ewes present, for faecal egg counts (FECs) at an external laboratory (Ridgeway Research, Glos., UK). Faecal samples were analysed using the modified McMaster method where one egg counted represents 50 eggs per gram. At the start of the trial this was two pooled samples of ten faecal samples for each paddock. However, because several ewes were removed from the trial due to the loss of their lamb(s) one group dropped to less than 15 ewes and subsequently only one pooled sample of 10 faecal samples was collected from this group.

Herbage samples were also taken from each paddock to perform pasture larval counts on the day before the ewes were populated into the field (week -2), three weeks into lambing (week +3) and again in the week of shearing of the ewes (week +9) using the method described by Molento et. al, [12]. Pasture larval counts were carried out at an external laboratory (Ridgeway Research, Glos., UK).

Faecal samples were collected from the lambs from six weeks into lambing until the week of shearing of their dams (week +6 to week +9). A pooled sample of ten faecal samples was taken from each paddock of lambs and sent to an external laboratory (Ridgeway Research, Glos.) for FEC testing.

Each lamb was weighed within twenty-four hours of birth, again at shearing of the ewes and again at weaning.

### 2.3 Health monitoring and treatments

All ewes and lambs were under veterinary supervision and were treated for any illnesses as necessary. The necessity of treatment for parasites was based on the FECs obtained and clinical signs seen in both the ewes and lambs. The assessment of a heavy burden was based on the SCOPS manual [13]. SCOPs define a high FEC as greater than 750 epg for mixed worm species in the absence of *Haemonchus contortus* and a heavy burden of *Nematodirus battu*s in lambs as greater than 300epg [13]. Lamb *Nematodirus battus* burdens exceeded 300epg in paddocks during week +7 and all lambs were treated with a benzimidazole as soon as possible following this result. Benzimidazole resistance in strongyle species is present on this farm and as such this treatment will not have affected strongyle FECs in the study lambs.

### 2.4 Statistical analysis

The statistical software used for all analyses was SPSS version 23 (IBM®).

#### 2.4.1 Ewes

The mean and standard error of ewe strongyle FECs were calculated for each treatment group for all weeks of the trial. Non-strongyle species were excluded from analysis either because the treatments used were not licensed for the treatment of those species or because they were not passed to lambs via faecal contamination of pasture by their mothers. The FEC data was non-parametric and could not be transformed to meet assumptions of normality and heterogeneity of variance. A full factorial Generalized Model (GLZM) was carried out to test the effects of week, treatment group and their interaction on ewe worm burden. Model fit was examined using SPSS Goodness of Fit tests.

#### 2.4.2 Lambs

The mean and standard error of lamb strongyle FEC was calculated for each field for weeks 6, 7, 8 and 9 of the trial only. The FEC data was non-parametric and could not be transformed to meet assumptions of normality and heterogeneity of variance. Kruskal-Wallis tests were carried out to test the effects of week and treatment group on lamb worm burden.

Daily live weight gains (DLWGs) and their standard errors were calculated for the period from birth to shearing of the ewes, birth to weaning and shearing to weaning for each field. Kruskal-Wallis tests were carried out to test the effect of treatment group on lamb DLWG.

#### 2.4.3 Pasture

The mean strongyle pasture larval count and the standard error were calculated for each treatment group for each of the sampling times.

#### 2.4.4 Ethical statement

Ethical approval was not required for this practical on-farm based trial. The animals available on the commercial study unit were used and not subjected to any non-routine farming procedures that were not already part of an established flock health plan and were under veterinary supervision through the trial period. Samples were collected via non invasive means, so no deviation from normal behaviour was noted.

## 3 Results

### 3.1 Periparturient faecal egg counts (FECs) of ewes which have received different treatments prior to lambing

**Fig 1:**
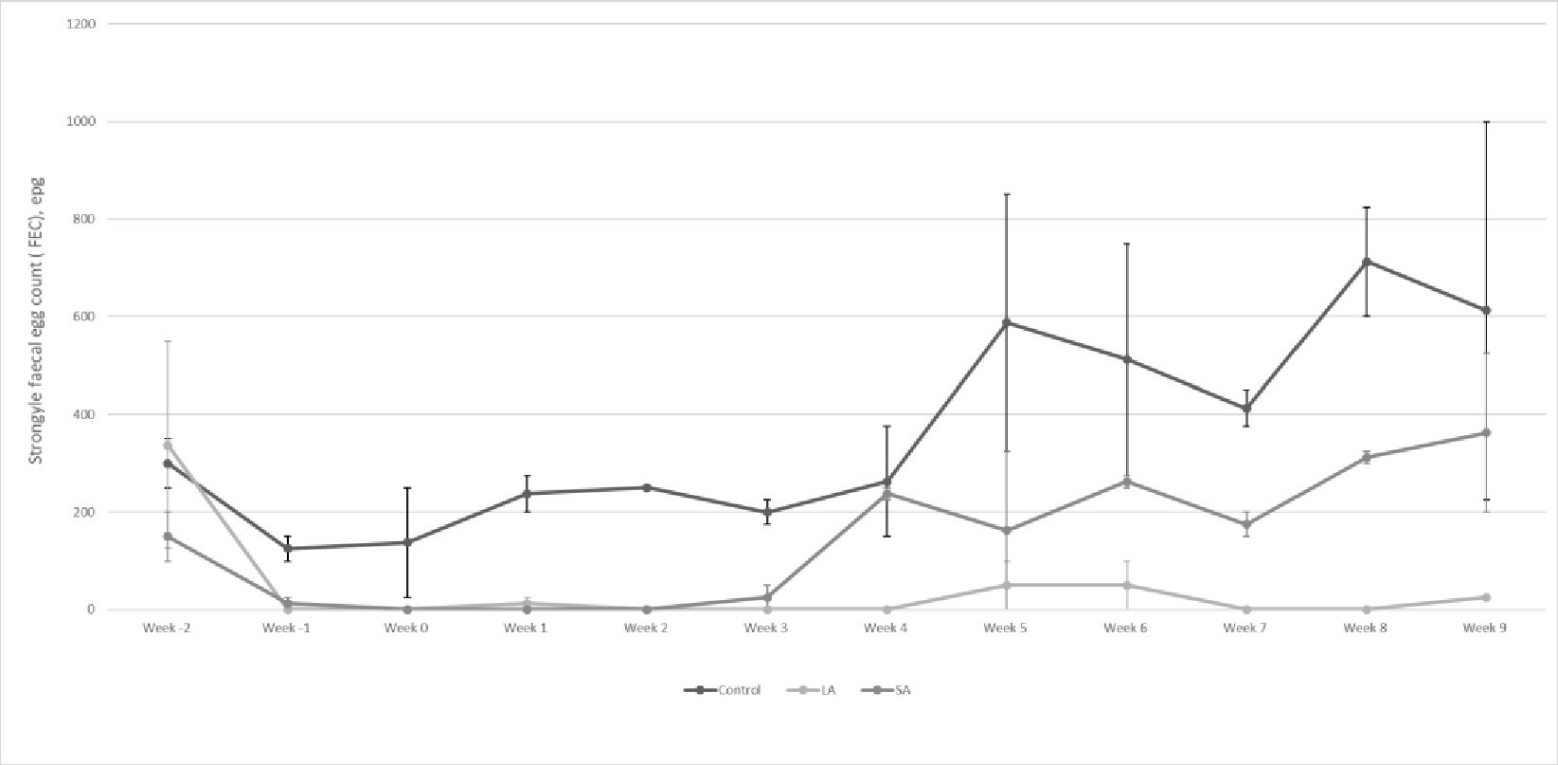
Mean strongyle FECs (+/- SE) of periparturient ewes receiving different treatments.

All paddock ewe groups had a FEC of greater than 100 in the week the study commenced with the highest FEC being 550 in ewe group C which received the long-acting wormer. The treatment groups Control, Long-acting wormer (LA) and Short-acting wormer (SA) had mean strongyle FECs of 300, 337.5 and 150 respectively. The effect of treatment (Wald Chi Square 110.123, df 2, p<0.001), week (Wald Chi Square 70.289, df 11, p<0.001) and the interaction of both (Wald Chi Square 52.825, df 22, p<0.001) were all found to have significant effect on FEC. Treatments were administered at the start of the study and by the next week mean FEC had dropped to 0 epg in the LA group and 12.5 epg in the SA group, whilst the control group’s FEC remained higher at 125 epg (Fig 1). The control group’s mean FEC steadily increased to 587.5 epg on week 5, and proceeded to vary between 412.5 and 712.5 over the rest of the study period (Fig 1). The LA group’s FEC remained 50 epg or below for the rest of the study period with a FEC of 0 epg for 7 of the 11 weeks post-treatment (Fig 1). The SA group’s mean FEC also dropped to zero from week 0 to week 2, it then increased to 25 in week 3 and then wavered between 162.5 and 362.5 for the following six weeks (Fig 1). Apart from week -1 to week 3 when LA and SA groups have similarly low FECs, there is little overlap between the three groups with the control group being consistently higher than the SA group which is consistently higher than the LA group from week 4.

### 3.2 Faecal Egg Counts (FECs) of lambs whose mothers have received different worming treatments prior to lambing

**Fig 2:**
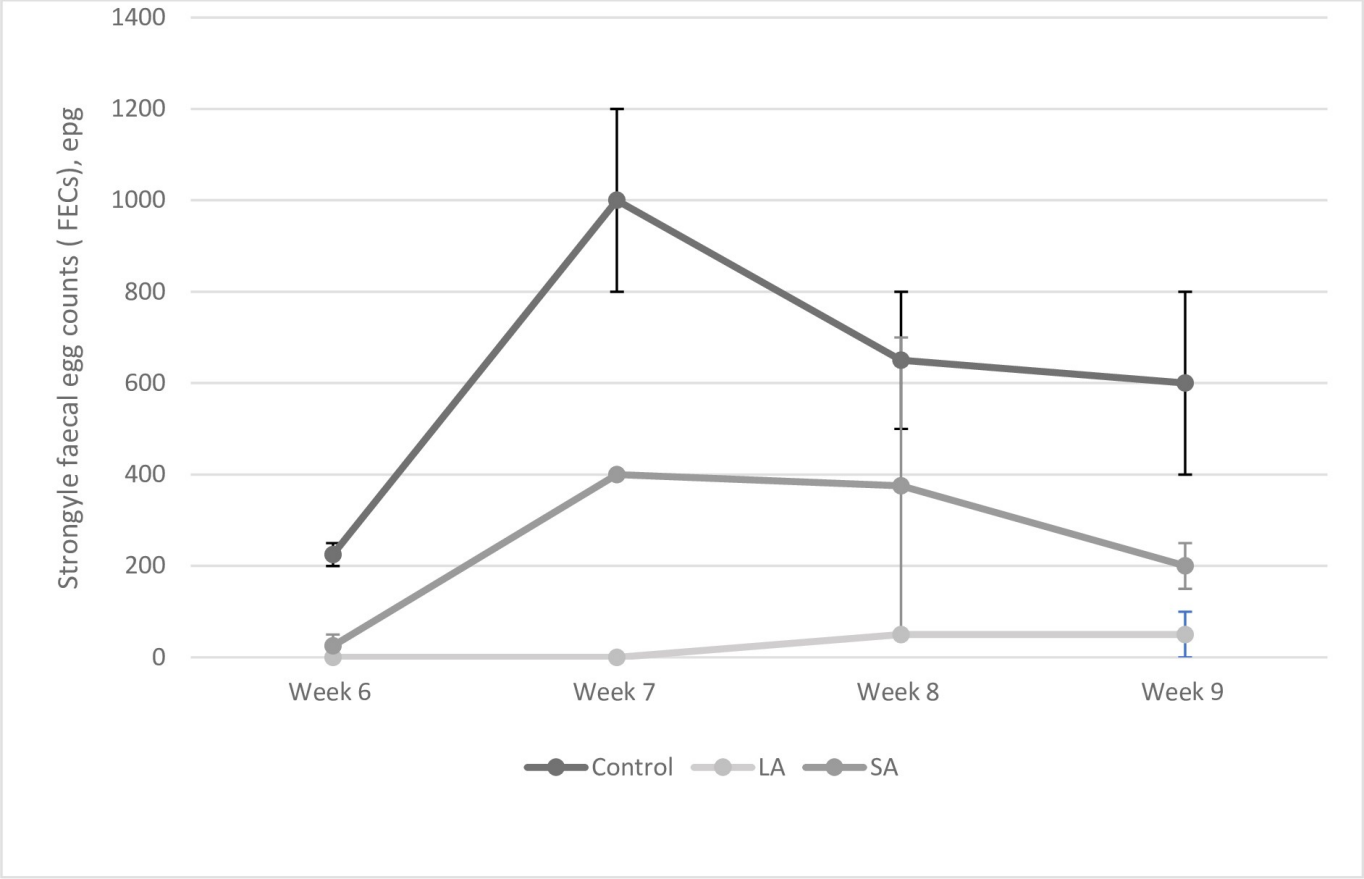
Mean FECs (+/-SE) of lambs from ewes which received different treatments prior to lambing from week 6 to week 9 after lambing began.

All lamb groups had low FECs of 250 or less in the first week of sampling with the highest FECs being in the two control groups A and B. Treatment group was found to have a significant effect on the lamb’s FEC (p = 0.001), while week did not (p = 0.284). Lambs from mothers who have not received any treatment had consistently higher FECs than those from ewes receiving treatment, with a peak of 1000epg in week 7 after which it decreases down to 600epg on week 9 (Fig 2). Both the LA group and the SA group had very low FECs on week 6 but the SA group had higher FECs throughout the rest of the period. The LA group’s FEC remained 50 epg or less (Fig 2).

### 3.3 Daily live weight gains of lambs whose mothers have received different worming treatments prior to lambing

**Fig 3:**
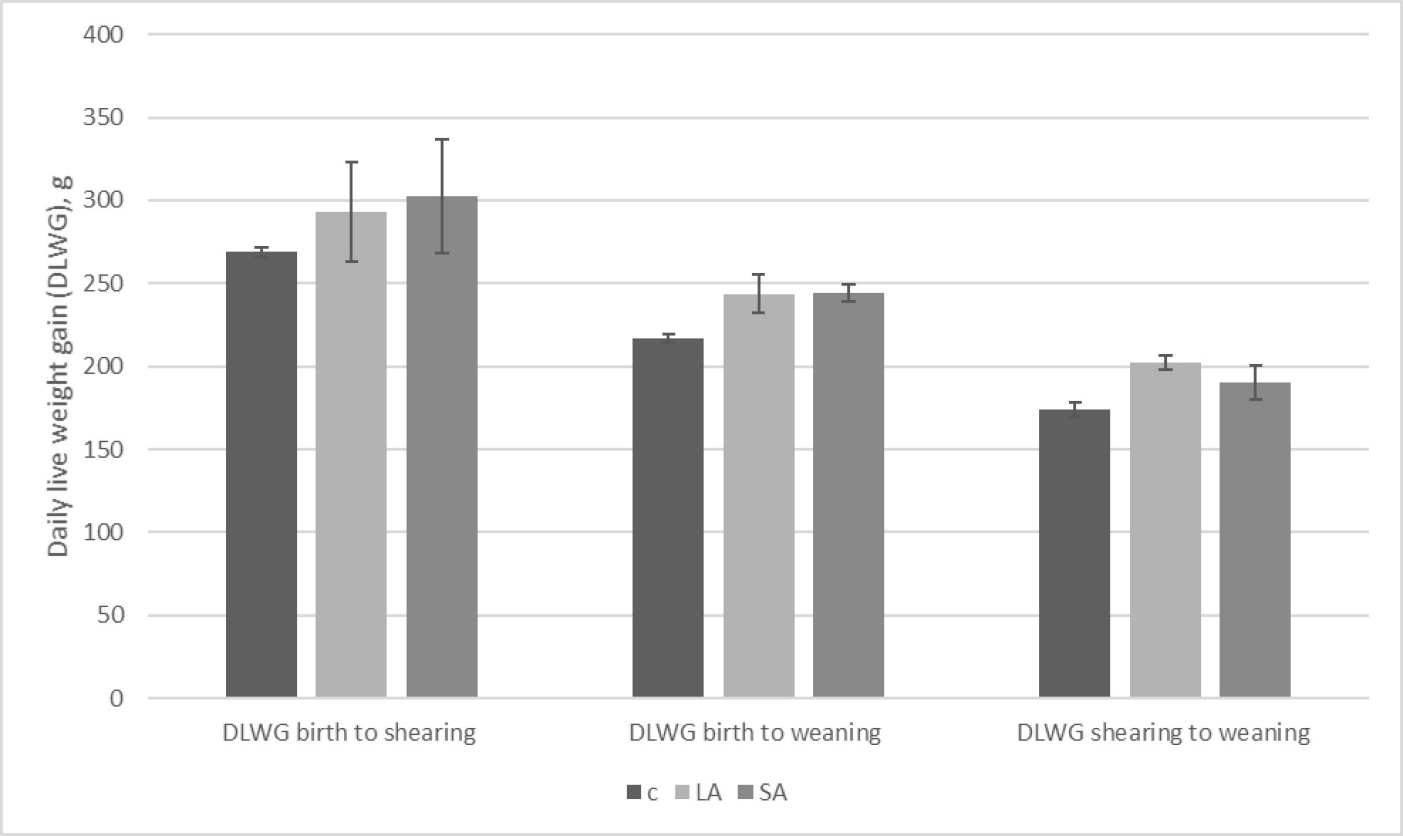
Mean DLWGs(+/-SE) for the control, LA and SA lamb groups from birth to shearing of the ewes, birth to weaning and shearing to weaning.

Differences in DLWG between treatment groups for the periods birth to shearing of the ewes, shearing to weaning and birth to weaning were not found to be statistically significant (p = 0.867, p = 0.156, p = 0.18 respectively). DLWG was highest from birth to shearing in the SA group while it was highest from shearing to weaning in the LA group (Fig 3). Over the entire period the SA group and LA group had almost the same DLWG (244g and 243.5g respectively). The control lambs had the lowest DLWGs (Fig 3).

### 3.4 Pasture larval counts of pasture grazed by ewes receiving different pre-lambing worming treatments

**Fig 4:**
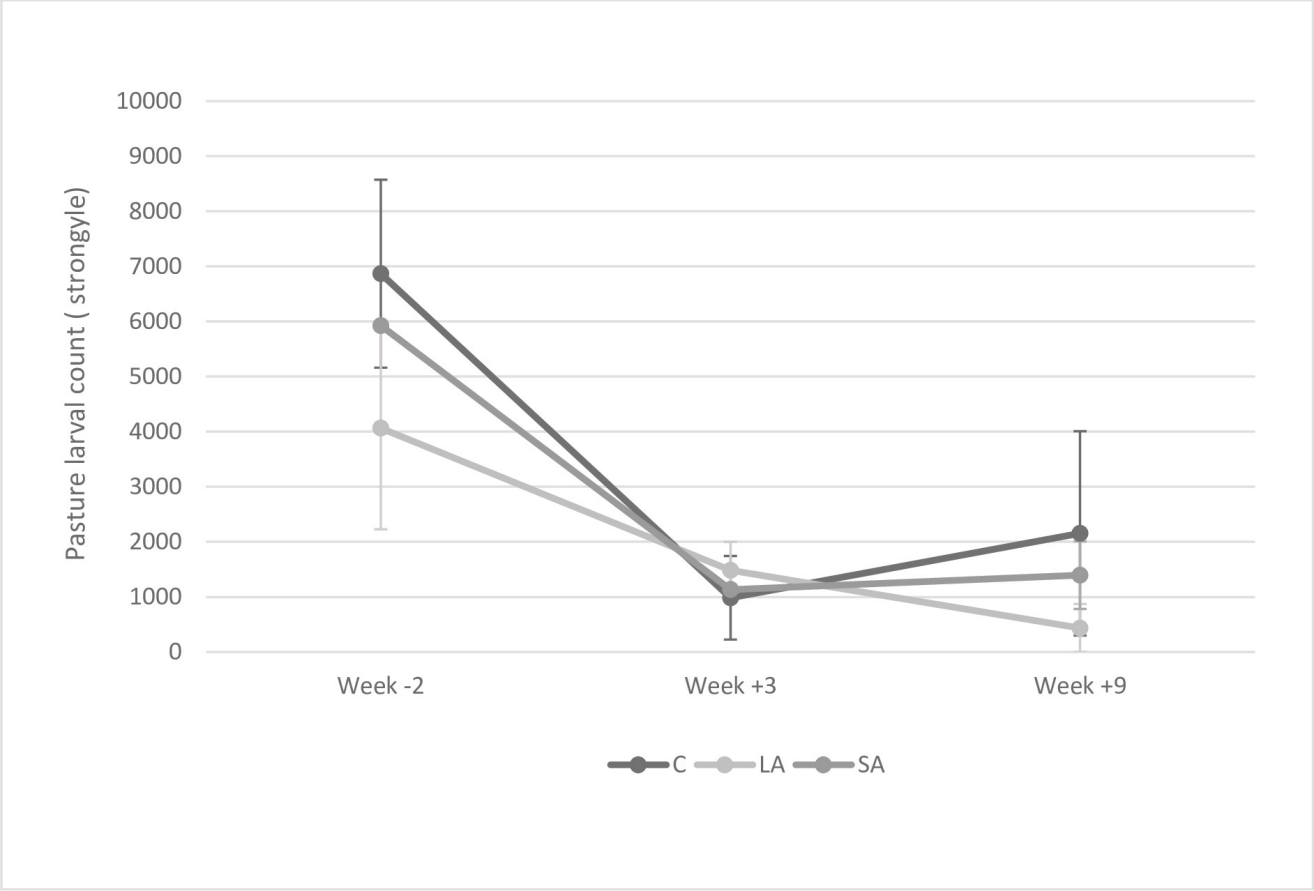
Mean strongyle pasture larval counts (+/-SE) for each treatment group at week -2, week 3 and week 9.

Strongyle pasture larval counts were initially very variable ranging from 4064 in the LA group to 6886 in the control group (Fig 4). At the second sample collection all groups’ strongyle pasture larval counts had dropped substantially to less than 1500 with little variation between the groups (Fig 4). Both the control group and SA group saw an increase in the strongyle PLC by week 9 while the LA group’s had continued to decline to 436 (Fig 4).

## 4 Discussion

### 4.1 Implications for parasite control

Ewes in this study received one of three treatments, a long-acting moxidectin dewormer, a short-acting doramectin dewormer or no deworming treatment, two weeks prior to the start of lambing. This was in order to investigate the effect on ewe and lamb worm burdens, pasture larval counts and lamb growth rates. The ewes receiving the long-acting moxidectin treatment maintained a reduction in FEC over the study period (11 weeks), in contrast to the group receiving short-acting anthelmintic, which had an increasing FEC from week 4 (Fig 1). Those ewes in the control group not receiving any treatment had a higher FEC than either the SA or LA treatment groups throughout the period studied, exceeding 400epg from week 5 onwards (Fig 1). This level of FEC is expected in ewes experiencing PPRI and not receiving an anthelmintic treatment [1]. The predominant pathogenic parasite on this farm at this time of year is *Teladorsagia cirumcincta*. The long-acting moxidectin product used is indicated as having a protective effect against *T*. *circumcincta* of 97 days [14]. This prolonged period of action is partly related to a longer elimination half-life of moxidectin in contrast to the avermectins [15]. The results of this study are comparable to those of [16] who found that ewes treated with a short-acting oral moxidectin excreted 3.5 times more *T*. *circumcincta* eggs than those treated with a long-acting moxidectin injection. The presence of ML-resistant parasites in the study by [16] must be noted as it is likely to have decreased the period of persistence of the oral moxidectin product which would be five weeks in the presence of a susceptible population, providing low or zero *T*. *circumcincta* egg counts for an eight-week period. The incomplete cover of the PPRI period by the short-acting wormer may lead farmers to repeat treatments of ewes while lactating in order to reduce infection of their lambs. Such repeated treatment may increase selection of resistant genotypes of gastro-intestinal parasites; a meta-analysis of research on factors affecting selection for anthelmintic resistance found the only significant risk factor was high frequency of treatment [17]. An additional disadvantage of repeated short-acting treatments in periparturient ewes is the need to gather and treat ewes with lambs at foot [16].

The lower ewe FECs in the LA group had a consequent impact on lamb FECs; those lambs born to ewes receiving long-acting wormer had the lowest FECs (Fig 2). The results of this study are in keeping with the findings of [18] who found lambs grazing with dams treated with long-acting moxidectin to have reduced FECs of 56% and 61% on two farms studied as compared to those whose dams were untreated [16] also found reduced FECs of lambs whose dams had been treated with long-acting moxidectin as opposed to a short-acting oral moxidectin. Reductions in lamb FECs could result in fewer or no anthelmintic treatments of the lambs, positively impacting on selection for anthelmintic resistance in the helminth population on a given farm, as well as reducing economic costs and labour time.

Lambs in the study by [18] were also found to have statistically significant higher DLWGs on one farm if their mothers had received a long-acting moxidectin treatment. In the present study, although the lambs whose mothers received long-acting moxidectin did have the highest DLWG of all the groups from shearing of the ewes to weaning it was not found to be statistically significant.

Pasture larval counts were relatively high at the start of the study but by the middle of the study period, strongyle pasture larval counts had decreased to less than 1500 in each treatment group. This may be attributed to particularly low rainfall in April in South-East England leading to desiccation of the strongyle larvae on pasture [19, 20]. However, the pasture larval counts rose in both the short-acting and control group in the latter half of the study, while the count decreased in the long-acting treatment group. This pasture larval count response corresponds with the increasing FECs of the SA and control groups after study mid-point while the FEC of the LA group remained low confirming that pasture larval counts are related to faecal excretion of helminth eggs. This has implications both for the infectivity of the pasture for the lambs grazing with their dams and for those animals which will graze this pasture the next year. Similarly, [16] found higher pasture larval counts of short sheath tail larvae, assumed to be *T*. *circumcincta*, on pasture grazed by ewes treated with short-acting wormer as compared to ewes treated with long-acting wormer.

### 4.2 Implications for the development of resistance

Although it is not advisable to use any one anthelmintic every year as a means of controlling PPRI, long-acting moxidectin could be useful as part of a flock parasite control programme in order to minimise the use of other treatments in that breeding year, for instance in the face of a high pasture burden which had built up over preceding years.

Wormers with persistent action have been found to be a risk factor for anthelmintic resistance selection [17]. It is suggested this is because all helminth eggs deposited on pasture at the end of the anthelmintic’s activity are derived from larvae which have survived exposure to anthelmintic [16]. This is disputed by some who suggest that helminths are exposed to lethal drug concentrations for longer periods when a long-acting moxidectin is used, therefore resistance is only selected for in the latter weeks of cover when the concentration within the animal dips beneath the lethal concentration, similar to short-acting wormers [16]. Research has shown that the terminal slope of serum concentrations is close to parallel for both the dose rate of short-acting and long-acting moxidectin, indicating that parasites would not be exposed to sub-lethal doses for any longer when the long-acting anthelmintic is administered [18].

Anthelmintics are likely to select more strongly for resistance when given to immune animals or those which regain their immunity while the product is still active, as well as during the ‘tail’ phase of anthelmintic activity when new larvae are ingested post-treatment [21, 22]. The ‘tail’ phase should not coincide with the recovery of immunity in the ewe. It is therefore important that the long-acting wormer is given to the ewe sufficiently long before lambing so that the effects are no longer present when the ewe’s immunity returns to its previous level in order to maintain an in refugia population. This must be particularly born in mind with breeds of sheep which have a shorter PPRI period such as Romneys [23]. Another means of achieving sufficient refugia population would be to only treat a proportion of the ewes. The SCOPS guidelines suggest treating 90% of ewes prior to lambing when using a long-acting formulation [13]. Guidelines on the responsible use of moxidectin have been created in conjunction with SCOPS in order to help delay the development of anthelmintic resistance.

### 4.3 Other implications of macrocyclic lactone use

The macrocyclic lactone group are partially eliminated in milk [15]. As such neither product used in this study is licensed for use in dairy ewes. [24] have shown that a macrocyclic lactone can be detected in the plasma of suckling lambs up to at least 20 days after it was administered to the dam and thus may select for resistant parasites within the lamb [25]. The transfer of anthelmintic via the milk to the lambs may also contribute to the reduction in FEC of lambs suckling dams receiving short-acting and long-acting macrocyclic-lactones [26].

### 4.4 Sustainable approaches to control of gastro-intestinal parasites in sheep

Combatting parasites and the associated anthelmintic resistance that has developed in recent years will require a multi-faceted approach, of which the use of a long-acting anthelmintic prior to lambing can be a part. Other approaches to decrease reliance on anthelmintics which can be incorporated into a sustainable plan for parasite control include genetic selection based on parasite resistance and resilience, grazing management, nutritional management, targeted selective treatment, effective quarantine procedures and grazing plants with anti-parasitic properties [13, 27–29]. Increasingly the focus of sustainable parasite management is on the maintenance of an *in refugia* population of worms, in order to dilute the impact of selection of resistance by anthelmintic use, largely through targeted treatment of those with high burdens and targeted selective treatment based on health and productivity impacts on the animal [30]. In order to implement refugia-based strategies it is necessary to accept a certain level of parasitism and thus consequent production losses in the short-term. One example of such a strategy might be to treat animals based on fecundity in the peri-parturient period [31]. The SCOPs guidelines incorporate a number of these practices [13]. Recent analysis of the effectiveness of these guidelines in managing parasites while reducing selection for resistance have shown a reduction in the quantity of anthelmintic used and slower development of anthelmintic resistance on farms which adhere to the SCOPS guidelines as opposed to those which follow traditional policies of parasite control while maintaining lamb productivity [32, 33].

## 5 Conclusions

This study has established the significant impact the use of long-acting moxidectin in ewes in the pre-lambing period can have on parasite burdens of ewes, their lambs and the resultant pasture contamination. A reduced number of treatments per season for ewes and lambs and a consequent reduction in the number of tail-phases due to reduction in overall anthelmintic use are among the benefits of its use.

Long-term strategies to find a balance in parasite control, maximising productivity as well as preserving current anthelmintic treatments effectiveness and that of those developed in the future, will require tailored, ever-evolving control plans for each farm with its individual conditions. The use of long-acting anthelmintics in the periparturient period can be incorporated into these strategies if they are used responsibly as a means of reducing pasture contamination. Future strategies will need to be practical to implement and cost-effective to maximise implementation by UK sheep farmers.

## Supporting information

S1 File (XLSX)

S2 File (XLSX)

S3 File (XLSX)
